# Grain Quality of Panicle Portions in Chalky and Low-Chalky Rice Cultivars

**DOI:** 10.1186/s12284-024-00751-7

**Published:** 2024-11-22

**Authors:** Stanley Omar PB. Samonte, Kimberly S. Ponce, Darlene L. Sanchez

**Affiliations:** Texas A&M AgriLife Research Center, Beaumont, TX 77713 USA

**Keywords:** Rice, *Oryza sativa* L., Yield, Quality, Stress, Chalkiness, Grain, Panicle

## Abstract

Carbohydrate stress reduces grain size and head rice percentage and increases the chalkiness in rice. This study aims to compare low and high-quality cultivars for their milled rice and chalky grain percentages, as well as grain size, in the top, middle, and bottom panicle portions. Low-chalky and chalky long-grain rice cultivars were grown at Beaumont in 2019 and 2022. Panicles were harvested, partitioned into top, middle, and bottom portions, and phenotyped for grain size, head rice percentage, and chalkiness. Grain area reduction percentage from top to middle panicle portions is higher in the low-chalky cultivars, Presidio and Kaybonnet. This could relieve the carbohydrate stress that leads to chalkiness. The rice cultivars were also genotyped for *Chalk5* and *OsPPDK*. The low-chalky cultivars had the same allele as the low-chalk Lemont for *Chalk5*. Presidio had a different allele for *OsPPDK* compared with the cultivars tested. Consistent with the genotyping result for *Chalk5*, Presidio and Kaybonnet had lower chalkiness than LaGrue and Leah. There was a positive correlation between the number of primary panicle branches and head rice percentage. The improvement in breeding efficiency for high grain quality requires phenotypic screening for a high number of primary panicle branches and for low chalky and partially chalky grain percentages.

**Supplementary Information:**

The online version contains supplementary material available at 10.1186/s12284-024-00751-7.

## Background

Grain quality traits are important factors affecting a rice cultivar’s acceptability by farmers and consumers. Rice cultivars should have a high and stable yield, the desired grain size, high whole-milled rice or head rice percentages (HRP), and low chalky grain percentage (CGP). Long, medium, and short grain types have length/width ratios greater than 3, between 2 and 3, and less than 2, respectively (Calingacion et al. 2014). Broken milled rice grains are kernels that are less than three-fourths the length of head rice.

Carbohydrate stress occurs during the grain-filling period. Chalkiness, measured as the ratio of the chalky area over the kernel area, occurs due to deficient amyloplast and protein development in the endosperm and increased interspaces between the loosely packed cells and amyloplasts (Lisle et al. 2000; Singh et al. 2003). Evaluation for chalkiness requires visual screening or grain analyzers to check the presence of white endosperm since the occurrence of chalky rice grains negatively impacts HRP. HRPs vary from one location to another and from year to year. High nighttime temperatures generally decrease HRP and grain size and increase chalkiness (Cooper et al. 2008; Lanning et al. 2011; Dou et al. 2018). Compared to low-temperature treatments, high temperature reduced HRP and grain widths without affecting lengths and thickness (Counce et al. 2005). In addition to high-temperature stress and varying field locations, N fertilization impacts grain chalkiness and HRP. Insufficient N fertilization results in lower grain filling rate, grain length and width, and increased chalkiness due to carbohydrate stress (Guo et al. 2022).

The minimum standards for acceptable rice in the United States are 55% HRP and 70% total milled rice percentages (TMRP). Presidio has been the standard check for grain quality in Texas, with 63% HRP, 72% TMRP, and 4.2% CGP. Trinity, its potential replacement, was released with a significantly higher grain yield. However, it has slightly lower HRP (62%) and TMRP (71%) percentages and slightly higher CGP (5.3%) (Samonte et al. 2022). In breeding programs, high-yielding advanced lines with low HRPs, high CGPs, or both are not advanced for further testing. Low HRPs and high CGPs are attributed to significant changes in the morphology and arrangement of starch granules as a result of a shortage in carbohydrate supply, insufficient grain-filling duration, and enhanced enzymatic activity involved in starch biosynthesis (Nagata et al. 2001; Du et al. 2023). The quality of rice grain in different portions of the panicle varies. Due to higher carbohydrate stress, grains in the middle and bottom panicle portions have lower grain quality than grains in the top portion. In the short grain premium quality rice cultivar Koshihikari, grains from lower panicle branches were smaller in length and width, and were chalkier than the middle and upper branches (Miyazaki et al. 2018). Chalky grains were smaller than non-chalky grains, even on the same branches, and their size was attributed to the lower number of cells or the inhibition of cell division during the early grain-filling stage. However, very limited studies have been conducted to characterize the chalkiness among panicle portions between chalky and non-chalky cultivars. This study, therefore, aimed to compare low-quality (LaGrue and Leah) and high-quality cultivars (Kaybonnet and Presidio) for grain size, milling quality, and chalkiness in the top, middle, and bottom panicle portions. The genotypic differences between low and high chalky groups were also determined using markers for *Chalk5* and *OsPPDK* genes. Results from this study will necessitate the determination of methods (e.g., breeding or agronomic) to improve the quality of the low-quality panicle portions, which eventually affects that of the entire panicle.

## Materials and Methods

### Genotypes and Field Experiments

Four tropical japonica long-grain cultivars from the USA with contrasting degrees of endosperm chalkiness were used in this study. Based on milled grain phenotypes, Kaybonnet (Gravois et al. 1995) and Presidio (McClung 2005) were classified as high-grain quality (low-chalk) rice, while LaGrue (Moldenhauer et al. 1994) and Leah (Trahan et al. 1982) were low-grain quality (chalky) rice. The four cultivars had short-stature heights of about 108, 94, 108, and 89 cm, respectively. Presidio reaches the heading stage the earliest, followed by LaGrue, Kaybonnet, and Leah, at about 4, 6, and 9 days later, respectively.

The field experiments were conducted at the Texas A&M AgriLife Research Center in Beaumont, Texas, using a randomized complete block design with three replications. Each plot consisted of six 2.5-m long rows with 28-cm row spacing. Seeds were planted on 13 May 2019 and 29 April 2022. In 2019, N fertilizer was applied at a rate of 59 kg N ha^− 1^,129 kg N ha^− 1^, and 47 kg N ha^− 1^ at the planting, permanent flood, and panicle differentiation stages, respectively. In 2022, N fertilizer was applied at a rate of 50 kg N ha^− 1^, 118 kg N ha^− 1^, and 56 kg N ha^− 1^ at the planting, permanent flood, and panicle differentiation stages, respectively. Total N applied may have been higher by 11 kg/ha in 2019 (235 kg ha^− 1^) than in 2022 (224 kg ha^− 1^), but this does not cause significant differences in grain yields (Atwill et al. 2021; Hou et al. 2021). The Beaumont Center maintains a weather station, and climatic data (daily temperature and solar radiation) used for this study were obtained from iAIMS Climatic Data website (Wilson et al. 2024b).

### Grain Quality Analyses

At maturity, 60 and 180 panicles per plot were randomly picked in 2019 and 2022, respectively. More panicles were picked in 2022 to increase the starting amount of rough rice analyzed for grain quality. Each panicle was partitioned into the top, middle, and bottom portions based on its number of primary panicle branches (NPPB). If the NPPB were divisible by three, then the top, middle, and bottom panicle portions would be allotted the same number of panicle branches. However, if after dividing by 3, there was a remainder of 1, then the remainder would be added to the bottom group, and it would have one more panicle branch than the middle and top panicle portions. If there was a remainder of 2, then the bottom and middle groups would have one more panicle branch than the top panicle portion. As an example, if NPPB = 15, then the top, middle, and bottom (T:M:B) panicle portions would have 5:5:5 panicle branches, respectively. If NPPB = 16, then the T:M:B panicle portions would have 5:5:6 panicle branches. Moreover, if NPPB = 17, then the T:M:B would be 5:6:6 branches.

Grain quality data were estimated for each portion. The starting rough rice amounts of each panicle portion for each of the three replications were 50 g in 2019 and 100 g in 2022. Total milled rice percentages were estimated from rice samples after dehulling and milling using a PAZ-1-DTA testing rice mill (Zaccaria). Broken rice percentages (BRPs) and head rice percentages were estimated after separating broken and whole milled rice portions using the CRZ 5 rice grader (Zaccaria). The S21 Rice Statistical Analyzer (TKD Tecnologia) was used to evaluate the head rice samples for grain length (GL), grain width (GW), length/width ratio (LWR), chalky area percentage (CAP), chalky grain percentage, partially chalky grain percentage (PCGP), and chalky + partial chalky percentage (CGP + PCGP). A rice grain was classified as a partially chalky grain if 25% < CAP < 50% and a chalky grain if CAP > 50%. The top-view grain area (GA) was estimated as GA = π(GL/2)(GW/2).

### Statistical Analyses

An analysis of variance (ANOVA) was conducted on the number of primary panicle branches, with year and block as random effects and cultivar as a fixed effect. ANOVA was conducted on 10 grain quality traits, with year and block as random effects, and cultivar and panicle portion as fixed effects. Each year was also analyzed separately, using chalky group, cultivar, and panicle portion as fixed effects and block as a random effect. Tukey’s Honest Significance Difference (HSD) test was used to compare treatment and interaction means. Linear contrasts were conducted to compare trait means of the low-chalky vs. high-chalky group. Correlation analysis was conducted to estimate the amount and significance of linear relationships between the number of primary panicle branches and the 10 grain quality traits. Path coefficient analysis (Li 1975; Williams et al. 1990) was conducted using the number of primary panicle branches as a third-order predictor variable, LWR and GA as second-order predictor variables, CGP and PCGP as first-order variables, and BRP and HRP as response variables. For each year, the data of these traits were standardized to a mean = 0 and a standard deviation = 1, then estimated for their path coefficients (p). JMP 13.2.1 software was used in all statistical analyses (JMP 2016).

### Molecular Marker Analyses for Chalkiness

To determine the genotypic differences for chalkiness between the low and high chalk groups, the four cultivars were genotyped for *Chalk5* (Li et al. 2014) and *OsPPDK* (Kang et al. 2005) using Lemont as the low-chalky check cultivar.

DNA of the rice cultivars was extracted following the ‘quick and dirty’ DNA extraction method (Collard et al. 2007). Polymerase chain reaction was performed on a T100 Thermal Cycler (Bio-Rad). The reaction was carried out with a final volume of 20 µl containing about 50 ng template DNA, 0.2 µmol L^− 1^ of each primer, and 1X *Taq* 2X Master Mix (NEB) using the following profile: 5 min at 94 ºC followed by 35 cycles of 30 s at 94 °C, 30 s at 55 °C, 30 s at 72 °C, and a final extension at 72 °C. The products were separated on 4% agarose gel and stained with ethidium bromide for visualizing DNA bands. The primers used are presented in Supplemental Table 1.

## Results

### Analyses of Variance

**Fig. 1:**
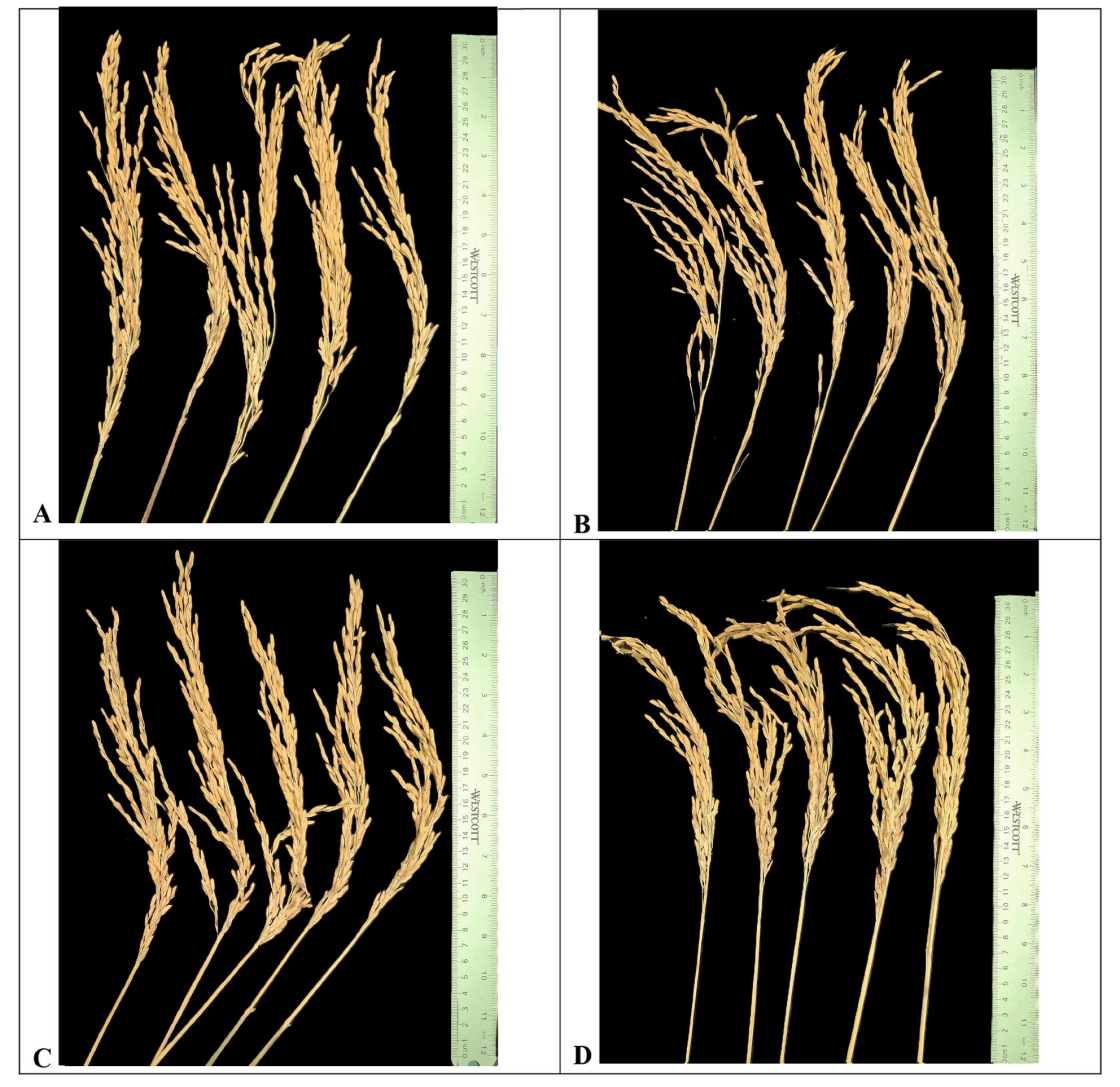
Images of panicles of the four long-grain rice cultivars evaluated in this study: **A**) Presidio, **B**) Kaybonnet, **C**) Leah, and **D**) LaGrue

Based on ANOVA, the number of primary panicle branches was significantly affected by year and variety x year interaction but not by cultivar (5% level). NPPB was highest in LaGrue and Kaybonnet in 2022, highest in Presidio in 2019, and significantly less in Leah compared to Kaybonnet, LaGrue, and Presidio in both years (Table 1; Fig. 1). NPPB was higher in 2022, ranging from 10.9 to 14.6 with a mean of 13.6, while it ranged from 10.0 to 11.1 with a mean of 10.7 in 2019.

**Table 1:** Number of primary panicle branches of four rice cultivars in Beaumont, Texas, in 2019 and 2022

Cultivar	No. of Primary Panicle Branches ^a^	
	2019	2022
Presidio	11.1b	14.4a
LaGrue	11.0b	14.6a
Kaybonnet	10.8b	14.6a
Leah	10.0c	10.9b

^a^ Means followed by the same letter are not significantly different based on Tukey’s HSD test at the 0.05 probability level

ANOVA showed significant variety x year interactions affecting 6 of the 10 grain quality traits (TMRP, GL, LWR, CAP, CGP, and CGP + PCGP) evaluated in this study (Table 2). Year significantly affected three traits (HRP, PCGP, and CGP + PCGP), while cultivar significantly affected HRP, GW, and GA. Panicle portion significantly affected GW, GA, HRP, and PCGP.

**Table 2:** Analyses of variance of grain quality traits estimated from rice samples harvested from Beaumont, Texas, in 2019 and 2022

Source of Variation	DF	Total Milled Rice			Head Rice Percentage			Grain Length				Grain Width			Grain Length/Width Ratio			
		SS	Prob > F	% Exp.^a^	SS	Prob > F	% Exp.	SS	Prob > F		% Exp.	SS	Prob > F	% Exp.^a^	SS		Prob > F	% Exp.
Year, Y	1	395.6	0.0801	30.5	996.1	0.0224*	10.9	0.242	0.2540		8.9	0.0333	0.7146	5.5	0.342		0.0649	23.5
Block[Y]	4	63.8	0.1567	4.9	59.6	0.3466	0.7	0.113	0.2997		4.1	0.0009	0.7110	0.2	0.024		0.4068	1.7
Variety, V	3	78.4	0.7433	6.0	6897.1	0.0054**	75.5	0.581	0.3492		21.3	0.5070	< 0.0001**	84.3	0.654		0.1027	44.9
V x Y	3	179.7	0.0098*	13.8	153.9	0.1859	1.7	0.357	0.0224*		13.1	0.0007	0.9607	0.1	0.124		0.0001**	8.5
Portion[Y, V]	16	180.3	0.2812	13.9	453.5	0.0208*	5.0	0.451	0.2641		16.5	0.0407	< 0.0001**	6.8	0.050		0.9194	3.4
Error	44	401.4		30.9	570.9		6.3	0.984			36.1	0.0187		3.1	0.262			18.0
Total		1299.1		100.0	9131.0		100.0	2.728			100.0	0.6014		100.0	1.456			100.0
**Source of Variation**	**DF**	**Grain Area**			**Chalky Area Percentage**			**Chalky Grain Percentage (CGP)**				**Partially Chalky Grain Percentage (PCGP)**			**CGP + PCGP**			
		**SS**	**Prob > F**	**% Exp.** ^a^	**SS**	**Prob > F**	**% Exp.**	**SS**	**Prob > F**		**% Exp.**	**SS**	**Prob > F**	**% Exp.** ^a^	**SS**		**Prob > F**	**% Exp.**
Year, Y	1	0.034	0.7507	0.1	974.2	0.1192	14.3	308.5		0.0739	28.4	956.6	0.0187*	48.5		2371.1	0.0156*	44.1
Block[Y]	4	0.349	0.2896	1.2	282.0	0.0056**	4.1	73.5		0.0012**	6.8	10.7	0.7719	0.5		134.3	0.0453*	2.5
Variety, V	3	21.458	0.0115*	73.9	4016.4	0.0797	58.9	385.0		0.2023	35.4	448.0	0.1204	22.7		1599.7	0.1264	29.7
V x Y	3	0.803	0.3206	2.8	620.7	< 0.0001**	9.1	132.6		< 0.0001**	12.2	97.0	0.0868	4.9		360.9	0.0091**	6.7
Portion[Y, V]	16	3.391	0.0014**	11.7	184.9	0.7870	2.7	38.8		0.7601	3.6	197.7	0.0289*	10.0		354.9	0.0713	6.6
Error	44	2.982		10.3	735.8		10.8	148.6			13.7	262.6		13.3		556.4		10.3
Total		29.017		100.0	6814.0		100.0	1087.0			100.0	1972.7		100.0		5377.4		100.0

^a^ Amount of variation explained by the source or factor

* Significant at the 0.05 probability level

** Significant at the 0.01 probability level

The genotype effect explained the majority of the variation in GW (84%), HRP (76%), GA (74%), CAP (59%), LWR (45%), CGP (35%), and GL (21%). In comparison, the year effect explained the majority of the variation in PCGP (49%), CGP + PCGP (44%), and TMRP (31%) (Table 2). Traits that panicle portion explained at least 5% of its variation were GL (17%), TMRP (14%), GA (12%), PCGP (10%), GW (7%), CGP + PCGP (7%), and HRP (5%).

### Weather during the Vegetative and Reproductive Stages

**Fig. 2:**
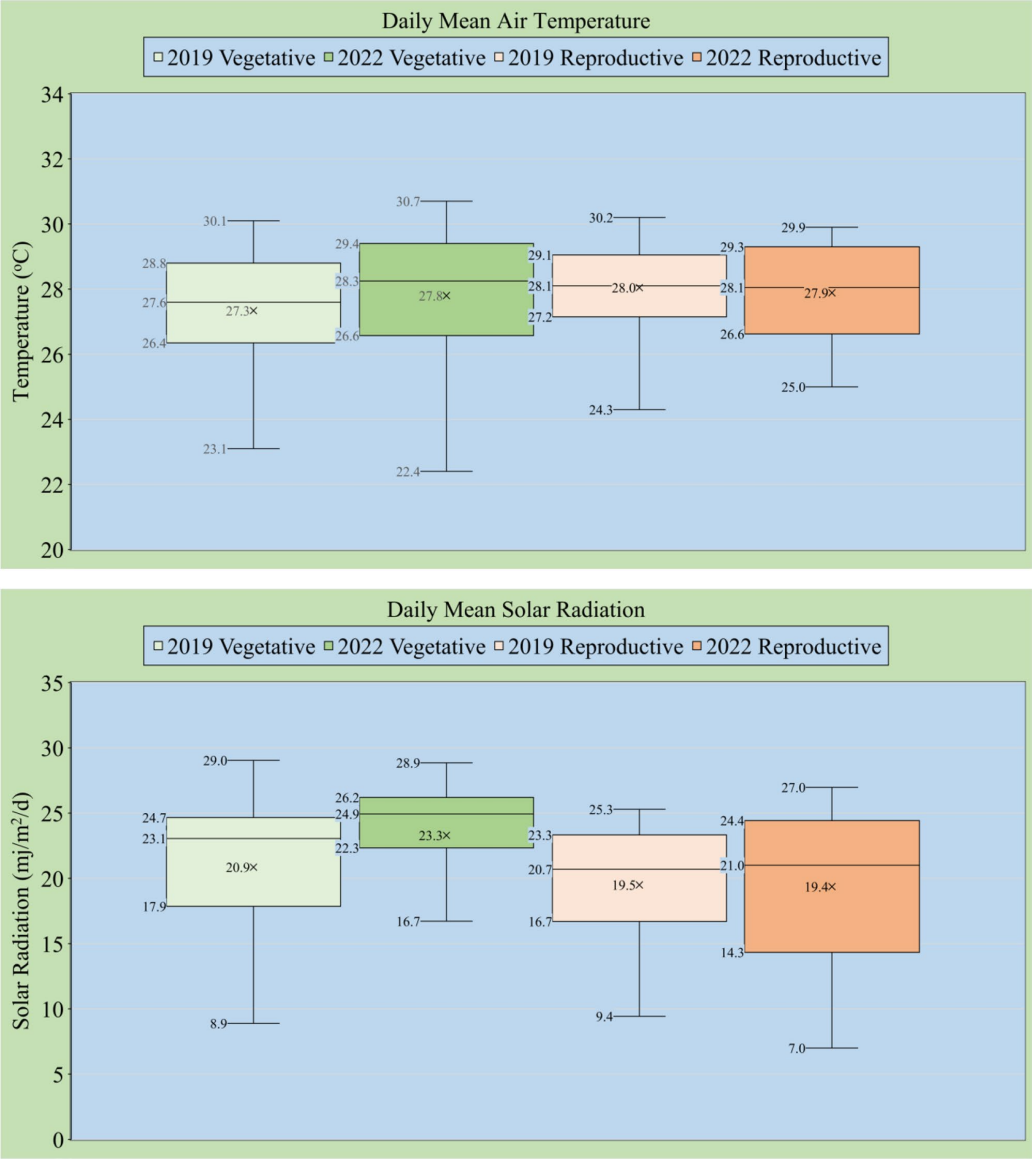
Box and Whisker plot of daily mean temperature (top) and solar radiation (bottom) during the vegetative and reproductive stages of four rice cultivars in Beaumont, Texas, in 2019 and 2022

During the seedling to heading period, the mean daily temperature was slightly higher in 2022 (27.8 °C) than in 2019 (27.3 °C) due to higher mean solar radiation, 23.3 vs. 20.9 mj/m^2^/d, respectively (Fig. 2). During the heading to maturity period, 2019 and 2022 were similar in mean daily temperatures (28.0 and 27.9 °C, respectively), mean daily maximum temperatures (33.8 and 33.0^o^C, respectively), and mean daily minimum temperatures (24.0 and 24.0^o^C, respectively). Mean solar radiation amounts were also similar at 19.5 and 19.4 mj/m^2^/d, respectively.

### Grain Quality of Rice Panicle Portions

#### Phenotypic and Genotypic Differences between Low and High Chalky Groups

**Fig. 3:**
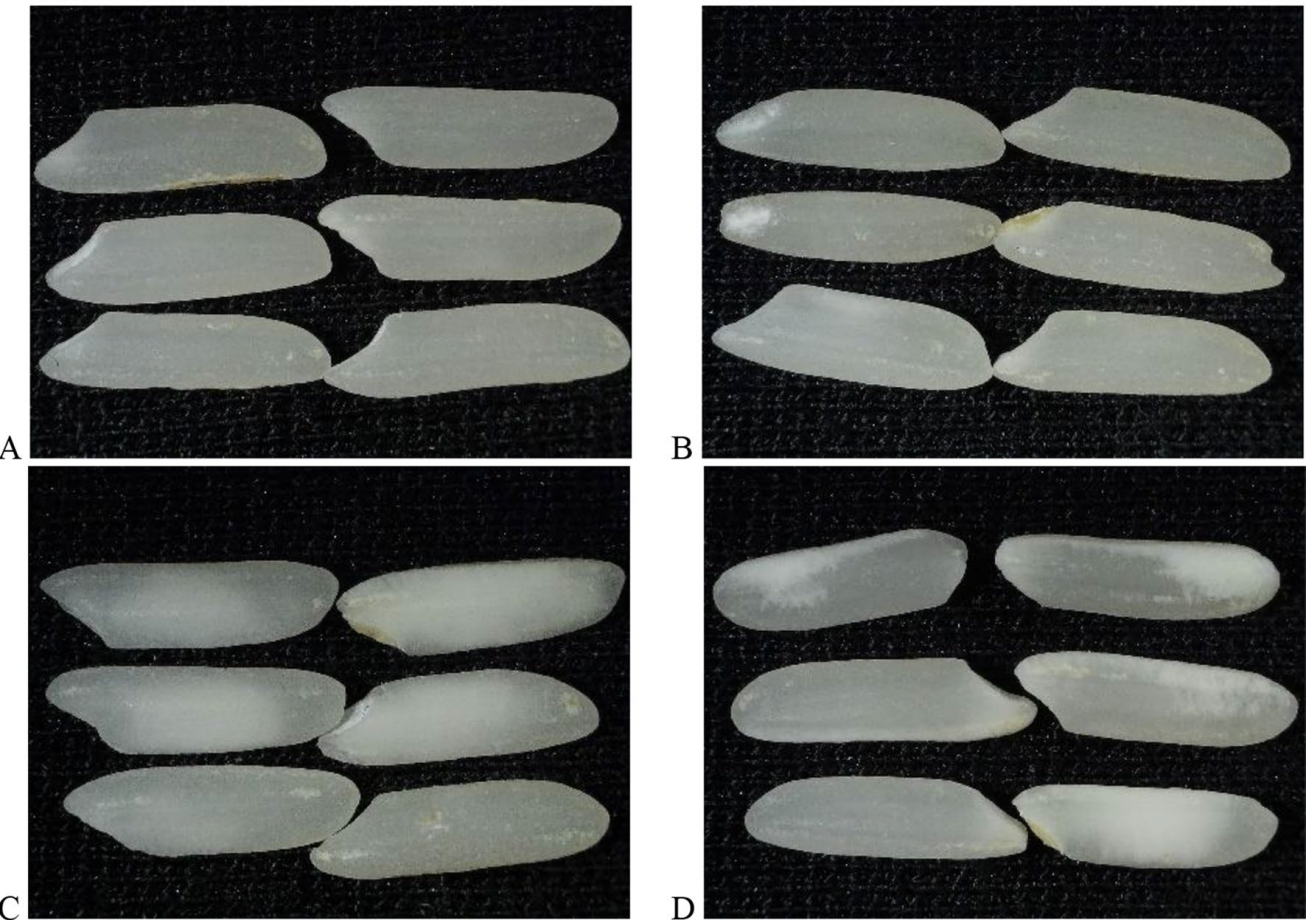
Milled rice kernels of rice cultivars evaluated: **A**) Presidio, **B**) Kaybonnet, **C**) Leah, and **D**) LaGrue

The means of grain quality traits of top, middle, and bottom panicle portions of four rice cultivars representing two rice chalky groups in 2019 and 2022 are presented in Table 3. The cultivars under the high-chalky (Leah and LaGrue) group had significantly higher CAP, CGP, PCGP, and CGP + PCGP in both years than the low-chalky group (Kaybonnet and Presidio) (Fig. 3).

**Table 3:** Grain quality of panicle portions of four rice cultivars in two chalky groups in Beaumont, Texas, in 2019 and 2022

Trait	Year	Panicle Portion	Rice Cultivar^a^				Chalkiness Group^b^	
			Kaybonnet	Presidio	Leah	LaGrue	Low	High
Chalky Area Percentage (%)	2019	Top	14.6c	17.7c	22.9bc	33.5a	16.2a	28.2ab
		Middle	14.1c	19.0c	22.4c	38.3a	16.5a	30.3a
		Bottom	13.3c	18.1c	17.9c	32.7ab	15.7a	25.3b
		Mean^c^	14.0c	18.2bc	21.0b	34.8a	16.1b	27.9a
	2022	Top	23.8bcde	22.6cde	37.6ab	37.9a	23.2a	37.8a
		Middle	18.5e	22.6cde	38.2a	35.9abcd	20.6a	37.0a
		Bottom	17.4e	22.3de	39.1a	36.7abc	19.9a	37.9a
		Mean	19.9b	22.5b	38.3a	36.8a	21.2b	37.6a
Chalky Grain Percentage, CGP (%)	2019	Top	0.21d	0.58d	1.05d	3.65bc	0.39a	2.35b
		Middle	0.22d	1.32 cd	1.50bcd	6.80a	0.77a	4.15a
		Bottom	0.22d	0.89d	0.79d	3.83b	0.56a	2.316b
		Mean	0.22b	0.93b	1.11b	4.76a	0.57b	2.94a
	2022	Top	3.46abc	2.89bc	9.15ab	9.82ab	3.17a	9.49a
		Middle	1.28c	3.34abc	10.42a	8.08abc	2.31a	9.25a
		Bottom	1.30c	3.27abc	9.69ab	8.05abc	2.28a	8.87a
		Mean	2.01b	3.16bc	9.75a	8.65a	2.59b	9.20a
Partially Chalky Grain Percentage, PCGP (%)	2019	Top	1.42e	3.74cde	2.49de	9.05ab	2.58b	5.77b
		Middle	2.00de	6.36bc	3.88cde	12.09a	4.18a	7.98a
		Bottom	1.25e	5.17 cd	3.44cde	11.01a	3.21ab	7.23a
		Mean	1.56d	5.09b	3.27c	10.72a	3.32b	6.99a
	2022	Top	14.86ab	12.80ab	13.52ab	15.48a	13.82a	14.50a
		Middle	8.06ab	13.74ab	13.78ab	15.10a	10.90a	14.44ab
		Bottom	5.41b	12.03ab	12.35ab	12.24ab	8.720a	12.29b
		Mean	9.44b	12.86ab	13.22ab	14.28a	11.15b	13.74a
CGP + PCGP (%)	2019	Top	1.64d	4.35 cd	3.59 cd	13.67b	2.99a	8.63b
		Middle	2.27d	7.76c	5.67 cd	19.56a	5.01a	12.61a
		Bottom	1.50d	6.11 cd	4.32 cd	15.13ab	3.81a	9.73b
		Mean	1.80c	6.07b	4.53b	16.12a	3.94b	10.32a
	2022	Top	18.43abc	15.89abc	23.44a	25.65a	17.15a	24.54a
		Middle	9.47bc	17.18abc	24.61a	23.41ab	13.32a	24.00ab
		Bottom	6.80c	15.39abc	22.44ab	20.59abc	11.09a	21.51b
		Mean	11.56bc	16.15b	23.50a	23.22a	13.85b	23.35a
Total Milled Rice (%)	2019	Top	67.3a	68.9a	67.9a	74.8a	68.1a	71.4a
		Middle	68.7a	69.2a	68.2a	67.6a	68.9a	67.9a
		Bottom	68.4a	68.7a	68.2a	68.0a	68.5a	68.1a
		Mean^c^	68.1a	68.9a	68.1a	70.1a	68.5a	69.1a
	2022	Top	63.3a	67.0a	67.6a	63.6a	65.2a	65.6a
		Middle	63.9a	67.2a	66.9a	59.0a	65.5ab	63.0a
		Bottom	62.7a	65.8a	64.1a	58.4a	64.3b	61.2a
		Mean	63.3ab	66.7a	66.2a	60.3b	65.0a	63.3a
Head Rice (%)	2019	Top	60.8a	60.0a	32.3d	51.8ab	60.4a	42.0a
		Middle	62.2a	63.6a	36.5 cd	42.6bcd	62.9a	39.6a
		Bottom	62.5a	62.4a	43.0bcd	45.4bc	62.5a	44.2a
		Mean	61.8a	62.0a	37.3c	46.6b	61.9a	41.9b
	2022	Top	53.6a	51.3a	34.5b	38.4b	52.4a	36.5a
		Middle	55.0a	55.7a	36.6b	37.2b	55.4a	36.9a
		Bottom	52.8a	53.8a	32.7b	32.3b	53.3b	32.5a
		Mean	53.8a	53.6a	34.6b	35.9b	53.7a	35.3b
Grain Length (mm)	2019	Top	6.19ab	6.22ab	6.41a	6.19ab	6.19a	6.30a
		Middle	5.94ab	6.10ab	6.38ab	6.15ab	6.02ab	6.27a
		Bottom	5.84b	6.07ab	6.46a	6.03ab	5.95b	6.24a
		Mean	5.98b	6.13b	6.42a	6.12b	6.05b	6.27a
	2022	Top	6.30a	6.41a	6.32a	6.44a	6.36a	6.38a
		Middle	6.16a	6.24a	6.30a	6.28a	6.20b	6.29ab
		Bottom	6.22a	6.18a	6.26a	6.22a	6.20b	6.24b
		Mean	6.22a	6.28a	6.29a	6.32a	6.25a	6.30a
Grain Width (mm)	2019	Top	1.87f	1.92d	2.10a	1.97c	1.89a	2.03a
		Middle	1.82f	1.89def	2.05b	1.91de	1.85b	1.98b
		Bottom	1.80 g	1.88ef	2.02b	1.89def	1.84c	1.96c
		Mean	1.83d	1.90c	2.06a	1.93b	1.86b	1.99a
	2022	Top	1.79de	1.88bc	2.03a	1.92b	1.84a	1.97a
		Middle	1.77e	1.85bcd	2.01a	1.89bc	1.81ab	1.95ab
		Bottom	1.77e	1.83cde	2.01a	1.86bcd	1.80b	1.93b
		Mean	1.78d	1.85c	2.01a	1.89b	1.82b	1.95a
Grain Length/Width Ratio	2019	Top	3.30a	3.24a	3.05a	3.14a	3.27a	3.10a
		Middle	3.26a	3.24a	3.11a	3.21a	3.25a	3.16a
		Bottom	3.25a	3.22a	3.20a	3.18a	3.24a	3.19a
		Mean	3.27a	3.23ab	3.12b	3.18ab	3.25a	3.15b
	2022	Top	3.51a	3.41abc	3.12d	3.35c	3.46a	3.23a
		Middle	3.48ab	3.37bc	3.14d	3.32c	3.42a	3.23a
		Bottom	3.52a	3.38bc	3.12d	3.35c	3.45a	3.23a
		Mean	3.50a	3.38b	3.13c	3.34b	3.44a	3.23b
Top-view Grain Area (mm^2^)	2019	Top	9.05 cd	9.38c	10.55a	9.57bc	9.21a	10.06a
		Middle	8.51de	9.04 cd	10.28ab	9.25 cd	8.77b	9.77ab
		Bottom	8.25e	8.96cde	10.24ab	8.97cde	8.60b	9.61b
		Mean	8.60c	9.12b	10.36a	9.26b	8.86b	9.81a
	2022	Top	8.88 cd	9.48abc	10.06a	9.73ab	9.18a	9.89a
		Middle	8.57d	9.08bcd	9.94a	9.33abc	8.82b	9.63ab
		Bottom	8.63d	8.88 cd	9.88a	9.09bcd	8.76b	9.49b
		Mean	8.69c	9.15b	9.96a	9.38b	8.92b	9.67a

^a^ Within the 3 rows (top, middle, and bottom panicle portions) of each year of each trait, cultivar x panicle portion means followed by at least one same letter are not significantly different based on Tukey’s HSD at the 0.05 probability level

^b^ Within the 3 rows (top, middle, and bottom panicle portions) of each year of each trait of each chalkiness group, means of chalkiness group x panicle portion interactions followed by at least one same letter are not significantly different based on linear contrasts at the 0.05 probability level. The low chalkiness group consists of Kaybonnet and Presidio, while the high chalkiness group consists of Leah and LaGrue

^c^ Means, averaged across panicle portions, followed by at least one same letter, are not significantly different based on Tukey’s HSD at the 0.05 probability level. Tukey’s HSD was applied separately for the cultivar and chalkiness group means

Leah and LaGrue also had significantly lower HRP than Kaybonnet and Presidio in both years (Table 3). Kaybonnet was the least chalky (lowest CAP, CGP, PCGP, CGP + PCGP) among the four cultivars in both years, while either LaGrue or Leah was the chalkiest (Table 3). Furthermore, the HRPs of Kaybonnet and Presidio were significantly higher than those of Leah and LaGrue in both years.

#### Effect of Year and Number of Primary Panicle Branches on Chalkiness and Head Rice

Yearly means were higher in 2022 than in 2019 for CAP (29.4 vs. 22.0%), CGP (5.9 vs. 1.8%), PCGP (12.4 vs. 5.2%), and CGP + PCGP (18.6 vs. 7.1%), with the latter three being significantly different based on *t*-test at 0.05 probability. HRP was significantly lower in 2022 than in 2019 (51.9 vs. 44.5%).

TMRPs were not significantly different between years and among cultivars in 2019, but LaGrue had significantly lower TMRP than the other cultivars in 2022. Within each cultivar, there were no significant differences in TMRP among panicle portions in both years (Table 3).

#### Effect of Year and Panicle Portion on Chalkiness and Head Rice Percentage

With regard to the significant effects of year and panicle portions on PCGP, chalkiness in both the low and high chalky groups was highest in the middle panicle portions in 2019 and the top portions in 2022. PCGPs were lowest in both chalky groups in the top panicle portions in 2019 and in the bottom portions in 2022 (Table 3).

#### Grain Size Dimensions and Areas of Top, Middle, and Bottom Panicle Portions

In both years, GL was not significantly different between panicle portions within each cultivar. However, grains were longest in the top panicle portion in 7 of the 8 variety x year (VxY) combinations and were shortest in the bottom portion in 6 of the 8 VxY combinations (Table 3). This indicates a trend of decreasing GL from top to bottom panicle portions. In both the low and high chalky groups, GL decreased based on panicle portion, from top > middle > bottom.

The four cultivars in this study are classified as long-grain rice since their head grain LWR is at least 3.0. Within each cultivar, their LWR did not significantly differ between panicle portions (Table 3). There were significant differences among cultivars in GW, with Leah > LaGrue > Presidio > Kaybonnet. Panicle portion also had a significant effect on GW. Rice grains were widest in the top panicle portion in all 8 VxY combinations and narrowest in the bottom portion in 7 of 8 combinations. A decreasing GW trend was observed from top to bottom panicle portions. A similar decreasing trend in the top-view GA was observed from top to bottom panicle portions. The LWR did not show this trend, and the panicle portions were not significantly different from each other within each cultivar or chalky group (Table 3).

The panicle portion had a significant effect on the top-view grain area, which takes length and width into account simultaneously. In this study, top-view GA decreased between panicle portions at different percentages for the low and high chalky grain groups. In 2019, the high-chalky group’s GA decreased by 2.9% from top to middle panicle portions and by an overall 4.5% from top to bottom. In contrast, the low-chalky group had decreased by 4.8% from the top to middle panicle portions and by an overall 6.6% from top to bottom panicle portions. The same trend was observed in 2022, i.e., the GA reduction, especially from top to middle panicle portions, was greater in the low than the high-chalky group. In 2022, there was a decreasing trend in CGP + PCGP from top to bottom panicle portions in both the low-chalky and high-chalky groups; however, the reduction was greater in the low-chalky group (17.2% in the top portion and 13.3% in the middle portion) than in the high-chalky group (24.5% in the top portion vs. 24.0% in the middle portion).

### Correlation between Number of Primary Panicle Branches and Grain Quality

There was a beneficial negative correlation (*r* = -0.49), though nonsignificant, between the number of panicle branches and CAP in 2022, but this was not exhibited during the potentially lower-yielding year 2019 (Table 4). In addition, there was a consistent positive correlation between the number of primary branches and HRP (*r* = 0.55 in 2022 with *p*-value = 0.06 and a nonsignificant *r* = 0.39 in 2019).

**Table 4:** Correlation between the number of primary panicle branches and grain quality traits at Beaumont, Texas, in 2019 and 2022

Grain Quality Trait	Correlation Coefficients and Probabilities (in Parentheses) with No. of Primary Panicle Branches	
	2019	2022
Chalky Area Percentage	0.11 (0.7244)	-0.49 (0.1030)
Chalky Grain Percentage (CGP)	0.17 (0.5873)	-0.48 (0.1112)
Partially Chalky Grain Percentage (PCGP)	0.28 (0.3745)	-0.16 (0.6131)
CGP + PCGP	0.25 (0.4422)	-0.42 (0.1710)
Total Milled Rice Percentage	0.19 (0.5455)	-0.50 (0.0941)
Head Rice Percentage	0.39 (0.2118)	0.55 (0.0618)
Head Rice Length	-0.51 (0.0925)	-0.24 (0.4606)
Head Rice Width	-0.43 (0.1585)	-0.85 (0.0004)
L/W Ratio	0.08 (0.8081)	0.84 (0.0006)
Top-View Grain Area	-0.50 (0.0995)	-0.82 (0.0012)

### Path Analysis of Rice Grain Quality Traits

**Fig. 4:**
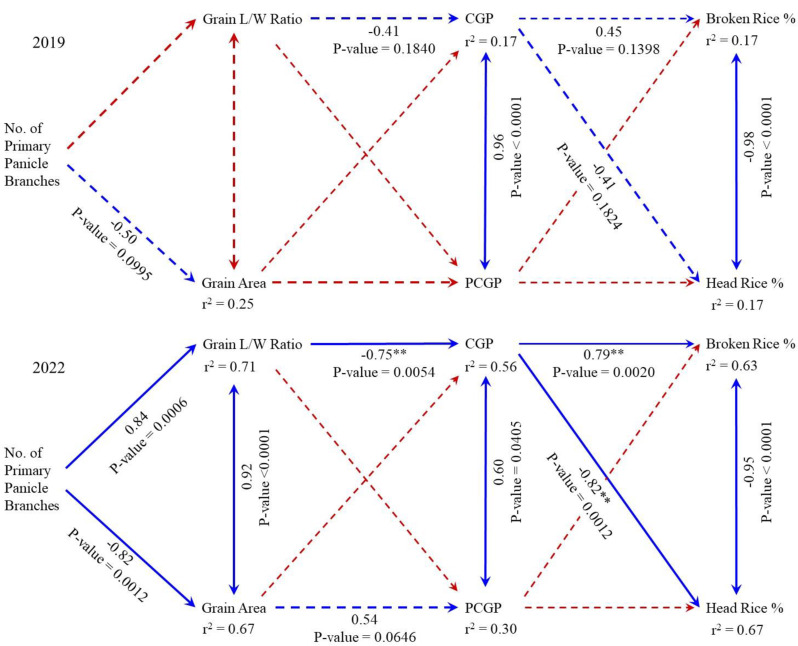
Path coefficients (single-arrowheaded lines) and correlation coefficients (double-arrowheaded lines) of rice grain quality traits at Beaumont, Texas, in 2019 and 2022. Solid blue lines are significant relationships at the 0.05 probability level, while broken red lines are nonsignificant

The path diagrams showing the estimates of direct effects (path coefficients) of predictor variables on response variables of rice at Beaumont in 2019 and 2022 are presented (Fig. 4). Similar trends observed in both years were the negative direct effect of the NPPB on GA, which was significant in 2022 at *p* = -0.82. There were also negative direct effects of LWR on CGP in both years, being significant in 2022 at *p* = -0.75. CGP had positive direct effects on BRP in both years, with the path coefficient *p* = 0.79 in 2022 being significant. Finally, CGP had negative direct effects on HRP in both years, with the path coefficient (*p* = -0.82) being significant in 2022. There was a significant direct effect of NPPB on LWR in 2022, but not in 2019.

From the path analysis results in 2022, the estimated direct effect of NPPB on CGP through LWR is *p* = -0.63, i.e., more panicle branches reduce chalkiness. Finally, the direct effect of NPPB, through LWR and CGP, was *p* = 0.52 on HRP and *p* = -0.50 on BRP, i.e., more panicle branches increase HRP and reduce BRP. Using the path analysis results from both years, the direct effect of LWR on HRP, through CGP, is *p* = 0.17 in 2019 and *p* = 0.62 in 2022, i.e., more elongated and slender rice increases HRP.

The significant correlations between CGP and PCGP were consistent across years. The expected significant negative correlation between BRP and HRP was also consistent across years. Not shown in the path diagrams were the nonsignificant correlations between total and whole rice or CGP in both years.

### Molecular Analysis for Chalkiness

**Fig. 5:**
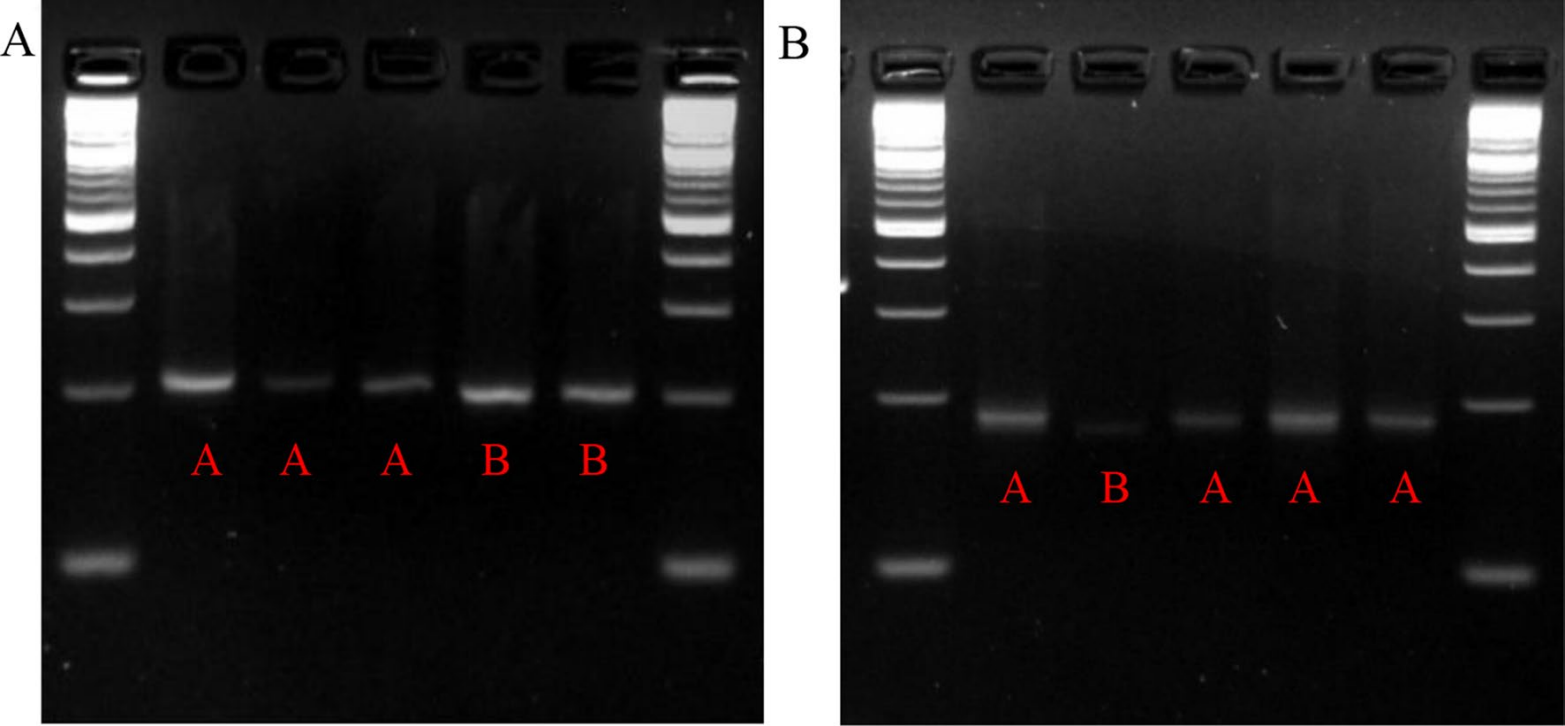
Genotyping results for (**A**) *Chalk5* and (**B**) *OsPPDK*. Bands from left to right are for Lemont, Presidio, Kaybonnet, Leah, and LaGrue

Based on molecular marker analysis for *Chalk5*, Kaybonnet and Presidio have the same allele as the low-chalky check cultivar Lemont (Fig. 5a). Marker analysis for *OsPPDK* showed that three cultivars under study, except Presidio, had the same allele as Lemont (Fig. 5b).

## Discussion

### Analyses of Variance

The number of primary panicle branches positively correlates with main culm panicle node number, plant height, LAI at heading, and biomass at heading (Samonte et al. 1998), i.e., larger plants tend to produce more panicle branches. In addition, NPPB is positively correlated with number of spikelets per panicle but negatively correlated with 100-grain weight (Samonte et al. 1998). A review paper indicated that the ideal panicle type in high-yielding cultivars would have more than 14 primary panicle branches (Xu et al. 2005). Although biomass and grain yield data were not obtained in this study, the higher NPPB in 2022 indicates a potentially higher biomass and grain yield than in 2019. These contrasting years provide the opportunity to analyze the effects of years on grain quality. The significant effects of year and variety x year interaction on NPPB suggest that subsequent analyses should be conducted separately for each year.

Knowledge of the factors affecting grain quality traits is essential in breeding high-yielding cultivars. Rice crop survey data show a negative relationship between grain yield and HRP (Wilson et al. 2024a), with the latter being negatively correlated to chalkiness, especially at high temperatures (Chen et al. 2017). This study indicates that cultivar was the main factor affecting most grain quality traits. When averaged across 10 traits, the cultivar effect explained 45% of the variation, while the year effect explained 21%. This emphasizes the importance of applying genetic markers in the breeding for desired high-quality lines, such as chalkiness, grain shape, amylose concentration, or aroma. The significance of the effect of panicle portion on some traits indicates that grain quality varies from top to bottom of the panicles. The identification of the panicle portion that exhibits the most variation for a trait is important for the rice breeder, as this indicates where grain quality screening should be applied. The relationships between changes in grain size and chalkiness may provide breeders with selection criteria to improve quality. The significant year and variety x year effects on several traits in this study support the analyses of results separately by year.

### Weather during the Vegetative and Reproductive Stages

Due to the similarity in mean daily temperatures (minimum, maximum, and mean) from heading to maturity in both years, temperature may have had minimal effects and may not be the main cause for the large grain quality differences between 2019 and 2022. High nighttime temperatures generally reduce head rice yields, grain size dimensions, and CGP (Cooper et al. 2008). However, a notable difference was the higher solar radiation amount during the seedling to heading period in 2022 than in 2019. This most likely contributed to the higher number of primary panicle branches and higher yield potential in 2022, which would have caused a higher carbohydrate demand during grain filling.

#### Effect of Year and Number of Primary Panicle Branches on Chalkiness and Head Rice

The observed low HRP was due to the observed high chalkiness (Table 3), although 2022 had possibly higher yield potential due to its higher number of primary branches. This trend was similar to those reported in the Texas rice crop surveys (Wilson et al. 2024a), i.e., Texas had a higher state mean grain yield (7,996 vs. 7,290 kg/ha) and a lower HRP (53.8 vs. 56.6%) in 2022 than in 2019. Comparing 2019 and 2022 Presidio production across Texas showed that 2022 had a higher mean grain yield (6,247 vs. 7,100 kg/ha) and lower HRP (54.7 vs. 35.0%) than 2019. This shows that, in general, 2022 was a higher yielding but lower grain quality year for rice in Texas than 2019.

#### Effect of Year and Panicle Portion on Chalkiness and Head Rice Percentage

During high-yielding years, such as in 2022, the demand for carbohydrates at the start of grain filling was high enough to cause stress resulting in higher chalkiness, unlike in 2019 when carbohydrate stress and chalkiness may have been highest when most of the middle panicle portion was being filled. In a related study, chalkiness decreased in order from top > middle > bottom panicle portions (Zhang et al. 2014), which is similar to the trend observed during the high-yielding year (2022) of this study.

#### Grain Size Dimensions and Areas of Top, Middle, and Bottom Panicle Portions

Rice spikelets undergo anthesis or self-pollination over three to several days, starting from the top of the panicle downwards to its base. The trend of decreasing GL is consistent with the expectation that the carbohydrate supply/demand ratio decreases as more grains are being filled after anthesis.

The consistent pattern of decreasing GL and GW from top to bottom panicle portions reflects the decreasing carbohydrate supply/demand ratio during grain filling. This is mainly caused by the increase in the number of grains simultaneously undergoing filling and the decrease in photosynthetic ability due to senescence and the translocation of nitrogen from leaves to the grain.

Grain filling rate has been observed to decrease rapidly during the middle and late stages (Dou et al. 2018). A greater reduction in the GA, especially from the top to middle panicle portions, could relieve the carbohydrate stress that causes the loosely packed starch granules, which are visible as chalky endosperm. As a result, there were no significant differences in CGP + PCGP among the top through bottom panicle portions in the low-chalky group in both years, unlike the high-chalky group, which showed significantly higher CGP + PCGP values in the middle panicle portions in 2019 (Table 3).

Several approaches have been suggested to improve or provide sufficient carbohydrate supply which ultimately reduces rice chalkiness. These include (1) increasing the nonstructural carbohydrates in leaf sheaths and culms reserved before heading (Samonte et al. 2001; Sakamoto and Matsuoka 2008) and (2) increasing the rate of N fertilizer, which increases the assimilation of carbohydrates in the functional leaves and improves grain filling rate in the middle and later stages of grain development (Guo et al. 2022).

### Correlation between Number of Primary Panicle Branches and Grain Quality

The negative correlation between the number of panicle branches and CAP in 2022 may indicate that a higher number of primary panicle branches may contribute to the reduction in grain chalkiness, especially when there is a high demand for carbohydrates during grain filling. The consistent positive correlation between the number of primary branches and HRP encourages the selection of rice panicles with a high number of panicle branches to have higher HRP. In a related study, more grains per panicle was negatively associated with quality, i.e., lower grain weight and larger variation in brown rice GL, GW, LWR, and CAP (Wang et al. 2008).

### Path Analysis of Rice Grain Quality Traits

The consistent negative direct effect of NPPB on GA implies that as more grains per panicle are produced, these are smaller, and in the case of 2022, the grains were more elongated and narrow. NPPB negatively correlates with 100-grain weight (Samonte et al. 1998), while a high number of grains per panicle is associated with larger variations in LWR (Wang et al. 2008). Furthermore, the path analyses implies that low-chalky cultivars with elongated and slender grains yielded significantly higher HRP than the chalky varieties. It also implies that rice breeders should not just select for low CGP, as currently practiced, but also for low PCGP.

The. nonsignificant correlations between total and whole rice or CGP in both years implies that a high TMRP does not necessarily result in high HRP and, therefore, low chalkiness. The path analysis provides rice breeders with selection criteria for improving grain quality traits. Overall, the long-grain rice breeder should select breeding lines with high NPPB, high LWR, low CGP and PCGP, and high HRP.

### Molecular Analysis for Chalkiness

The observed phenotypic difference in the grain chalkiness of the low and high chalky groups is further supported by the genotyping result for the *Chalk5* gene, with Presidio and Kaybonnet carrying the allele for low chalkiness similar to Lemont. Meanwhile, the *OsPPDK* allele of Presidio, which is different from all the cultivars tested, likely contributes to its low chalkiness apart from its *Chalk5* allele. *OsPPDK* is another gene that contributes to grain chalkiness by controlling carbon metabolism during grain filling (Kang et al. 2005). However, the marker used for this gene was not able to distinguish low and high chalky groups. This is likely because *Chalk5* is the major gene that contributes to the natural variation in rice grain chalkiness (Li et al. 2014).

## Conclusion

The differences in the chalkiness between the low and high chalky long-grain rice groups were mainly due to the allelic differences for *Chalk5*, with the low-chalky group having the same allele as the low-chalky check cultivar Lemont. Moreover, a significant difference in the percentage of grain area reduction across panicle portions was observed between the two groups. The greater reduction in the grain area, especially from the top to middle panicle portions, in the low-chalky cultivars is possibly due to a slow grain filling rate. This could relieve the carbohydrate stress that causes loose packing of starch granules, which ultimately appear as chalky endosperm. Path analysis revealed that the number of primary panicle branches has negative direct effects on chalky and partially chalky grain percentages and has a positive direct effect on head rice percentage. This implies that the efficiency of breeding for high-grain quality also requires phenotypic screening for high NPPB and low PCGP, in addition to low CGP.

## Electronic supplementary material

Below is the link to the electronic supplementary material.

Supplementary Material 1

## Data Availability

No datasets were generated or analysed during the current study.

## Abbreviations

CAP: Chalky Area Percentage
CGP: Chalky Grain Percentage
GA: Grain Area
GL: Grain Length
GW: Grain Width
HRP: Head Rice Percentage
LWR: Length/Width Ratio
NPPB: Number of Primary Panicle Branches
PCGP: Partially Chalky Grain Percentage
TMRP: Total Milled Rice Percentages
