# Supplementary material for: Grain Quality of Panicle Portions in Chalky and Low-Chalky Rice Cultivars
Source: Rice (N Y). 2024 Nov 22;17:71. doi: 10.1186/s12284-024-00751-7 (PMC11584826; doi:10.1186/s12284-024-00751-7)
Supplement: Supplementary file 1 — Supplementary Material 1 [file 12284_2024_751_MOESM1_ESM.docx]

**Supplemental Table 1.** Primers used in genotyping for *Chalk5* and *OsPPDK* genes.

| Gene | Forward Primer Sequence | Reverse Primer Sequence | Expected Product Size (bp) | Chromosome |
| --- | --- | --- | --- | --- |
| *Chalk5* | GTTTGCATCTCTCTTTGCTG | CAAGAGGAACTCATCTGTAG | 237 | 5 |
| *OsPPDK* | GCTTGAAACAAGCTAAAATACCG | CAGATTATTTGCTCACGCTATAAAA | 176 | 5 |
